# Women’s health: optimal nutrition throughout the lifecycle

**DOI:** 10.1007/s00394-022-02915-x

**Published:** 2022-05-25

**Authors:** Edith J. M. Feskens, Regan Bailey, Zulfiqar Bhutta, Hans-Konrad Biesalski, Heather Eicher-Miller, Klaus Krämer, Wen-Harn Pan, James C. Griffiths

**Affiliations:** 1 Wageningen University, Wageningen, The Netherlands; 2 Institute for Advancing Health Through Agriculture, Texas A&M University System, College Station, TX USA; 3 Centre for Global Child Health, Toronto, Canada; 4 Aga Khan University, Karachi, Pakistan; 5 University of Hohenheim, Stuttgart, Germany; 6 Purdue University, West Lafayette, IN USA; 7 Sight & Life, Basel, Switzerland; 8 Johns Hopkins University, Baltimore, MD USA; 9 Academia Sinica, Taipei, Taiwan; 10 Council for Responsible Nutrition-International, Washington, DC USA

**Keywords:** Ageing, Diet, Disparities, Life-expectancy, Nutrition, Women’s-health

## Abstract

Sex differences are an important consideration when researching and establishing policies for nutrition and optimal health. For women’s health, there are important physiologic, neurologic, and hormonal distinctions throughout the lifecycle that impact nutritional needs. Distinct from those for men, these nutritional needs must be translated into appropriate nutrition policy that aims to not only avoid overt nutritional deficiency, but also to promote health and minimize risk for chronic disease. Through a series of webinars, scientific experts discussed the advances in the understanding of the unique nutritional needs, challenges and opportunities of the various life stages for women across the life course and identified emerging nutritional interventions that may be beneficial for women. Nevertheless, there is concern that existing nutrition policy intended for women’s health is falling short with examples of programs that are focused more on delivering calories than achieving optimal nutrition. To be locally effective, targeted nutrition needs to offer different proposals for different cultural, socio-economic, and geographic communities, and needs to be applicable at all stages of growth and development. There must be adequate access to nutritious foods, and the information to understand and implement proven nutritional opportunities. Experts provided recommendations for improvement of current entitlement programs that will address accessibility and other social and environmental issues to support women properly throughout the lifecycle.

## Introduction

The 2030 agenda for sustainable development, adopted by the United Nations (UN), is the current cornerstone of many health policies [169]. The importance of this agenda has recently been stressed by the successful and widely attended Tokyo Food Systems Summit, in December 2021, which launched bold new actions to deliver progress on all 17 Sustainable Development Goals (SDGs) [170]. These SDGs relate to nutrition not only by *SGD2 Zero Hunger*, or *SDG3 Good Health and Wellbeing* but also *SDG5 Gender Equality* [69]. This is very important, as women are especially vulnerable regarding food insecurity in several life stages, such as pregnancy and frailty. As shown by, e.g., Dutch Hungerwinter studies, it is now well established that malnutrition in utero not only leads to low birth weights or less healthy babies but also impacts the occurrence of non-communicable diseases in later life [47, 161]. As such, healthy nutrition during an infant’s first 1000 days, and also including the mothers, is key to healthy populations in all cultures.

An often overlooked life stage in women’s nutrition is adolescence. Adolescents can be regarded as our future workforce and bearers of our next generation. Therefore, improving their health and development is crucial in shaping the health and wellbeing of this generation and the next and the next ad infinitum. During adolescence, numerous biological and psychosocial changes prompt the transition from childhood to adult life. It is a period of hormonal changes; the production of adrenal androgens increases, and the growth hormone and thyroid axes mature. In this period, 50% of the adult body weight and 15–25% of final height are gained [46]. In sum, adolescence is a period of rapid growth, and consequently, the energy and nutrient requirements increase. In addition, in this life stage, dietary patterns, physical activity, and eating behavior are heavily influenced by internal factors (such as attitudes, beliefs, perceived barriers, food preferences, self-efficacy, and biological changes), external factors (family, friends, fast food outlets, and social and cultural norms), and macro-systems (such as food availability, food production, distribution systems, mass media, and advertising) [136]. Thus, adolescents are nutritionally vulnerable because of the increased nutritional demands alongside the social adaptation to adulthood.

Often this period is referred to as the second window of opportunity, to catch up with linear growth [136], and in addition to the first 1000 days is a crucial nutrition-sensitive developmental stage. Under the umbrella of a program called ‘Ten-to-Twenty’, several academic research projects have recently been carried out at Wageningen University. The study in Mexico focused on adolescent girls and boys aged 12–19 years and showed that both a Western- and plant-based dietary pattern were simultaneously associated with overweight–obesity and at least one indicator of under-nutrition such as anemia [192]. In Ghana, time trends in anthropometry in adolescent girls aged 15–19 years showed that thinness and stunting had declined since 2003, but that the prevalence of overweight and obesity had increased by 40%, while anemia remained severe [11]. There is a double burden of malnutrition as evident in this vulnerable group, and becoming even more prominent, suggesting that obesity and anemia can co-occur in the same girls, which needs to be taken into account in dietary advice and nutrition programs. Indeed, an interdisciplinary study in Nepal among adolescent girls showed that thinness and anemia were negatively associated with adolescent girls’ aspirations in domains of fertility and education. Hence, multisectoral integrated policies and programs that improve adolescent nutritional status and diets have the potential to foster adolescent girls’ objectives and thereby increase their future potential [110].

Menopause is another female life stage with health and nutritional consequences. Many deleterious physiological changes take place around this time point due to hormonal changes. In earlier studies, we have seen that hormonal changes induce changes in body fat composition and fat distribution [96]. As a consequence, cardio-metabolic risk factors increase and the risk of diabetes and cardiovascular disease (CVD) reach patterns similar to what is observed in males. A sharp bone mineral density (BMD) downturn is also observed at the same time; traditionally, calcium and vitamin D are regarded as important nutrients in preventing reduction in BMD and preventing osteoporosis. However, other nutrients may also play a role. Osteoporosis occurred more often among women whose nutritional status was marginal on vitamin B12, magnesium, and poor phytonutrient intakes [51]. How women adjust their lifestyle during the menopausal period is critical for healthy aging. Data mining studies showed that dietary patterns associated with better prognosis of geriatric syndromes are in line with plant-based diet [38]. This study points at an emerging issue of concern in nutrition nowadays: the role of impact on the environment, i.e., not only considering human health but also planetary health [187]. Eating less meat and shifting to a plant-based diet may be a good solution for this, but the context needs to be taken into account; in regions with food insecurity and high prevalence rates of anemia and other micronutrient deficiencies, a careful trade-off needs to be made.

Such efforts should include informed decisions on which nutrients and which amounts are needed from the industry perspective. Additionally, given the differential use of prenatal dietary supplement (DS) products based on maternal age, education, race and ethnicity, income, and health insurance status, clinician efforts should focus on increasing access to prenatal DS, and to understand the barriers to their use in population subgroups. Knowledge dissemination strategies may also be needed at the public health level to reduce nutrient disparities.

## Hidden hunger: the case of Germany

Although it is hard to imagine that children live in food poverty in Germany, one of the richest countries, the limited data shows that this is indeed the case and it is obviously overlooked by policy makers; accordingly to the saying, “weil nicht sein kann, was nicht sein darf” (“what cannot be, may not be”) [116]. The scientific advisory board of the Ministry of Food and Agriculture^1^ presented the problem in an expert report from 2021 and called on policy makers to take the appropriate action. The consequences for inaction are for the children who may be affected with physical and cognitive developmental disorders that greatly reduce their chances of escaping the poverty trap.

### A healthy diet for children cannot be financed with the funds for nutrition from the unemployment benefits

In Germany, 21.3% (2.8 million) children and young people under 18 currently live in poverty. Most of these children (between 40 and 55% depending on the federal state) live in single-parent families and in most cases with two or more siblings. This means not only social and psychological problems, but also that a healthy diet can hardly be guaranteed for these children, a condition defined as food poverty [182].

The ‘wholefoods diet’ is a good example of a healthy diet that contains macro- and micronutrients in quantities that not only meet nutritional needs but also prevent diet-related diseases. In the Giessen Whole Food Nutrition Study (women only), this was compared with a typical mixed healthy diet, with monthly per person costs of 227 €/month for a wholefood diet and 259 €/month for a health mixed diet [113]. The ALGII (unemployment budget) standard for children is shown in Table 1.

**Table 1:** Standard requirement for basic support (2019)

Age	Total per month	Share of food, non-alc. Beverages (month)	Share of food, non-alc. Beverages (day)
Adolescents 14–17	322 €	151.57 €	4.98 €
Children 6–13	302 €	122.01 €	4.01 €
Children 0–5	245 €	85.87 €	2.82 €

The total amount must be used to finance the entire costs of living, including expenses for health care or clothing, etc. The share that can be used for food is fixed and the possibilities to buy food depend strongly on price increases in the food and non-food sector

The cost of a healthy diet is at least 25% higher than the available resources in all age groups. A qualitatively sufficient healthy diet, especially for children and adolescents, is consequently not affordable.

### Nutrition of children in families receiving unemployment benefits is often poor in essential micronutrients

Basically, this is a question of how the diet is composed. Ultimately, any micronutrient that is not sufficiently contained in the diet can be affected. In Germany, apart from rare exceptions, one will hardly see a severe nutritional deficiency with typical symptoms, such as rickets or scurvy. The inadequate supply of nutrients, especially micronutrients, results in what is termed ‘hidden hunger’. Hidden, because the deficiency does not reveal itself through typical symptoms, and as long as no typical clinical symptoms occur, it remains in many cases unclear which micronutrients are affected [22, 24].

**Fig. 1:**
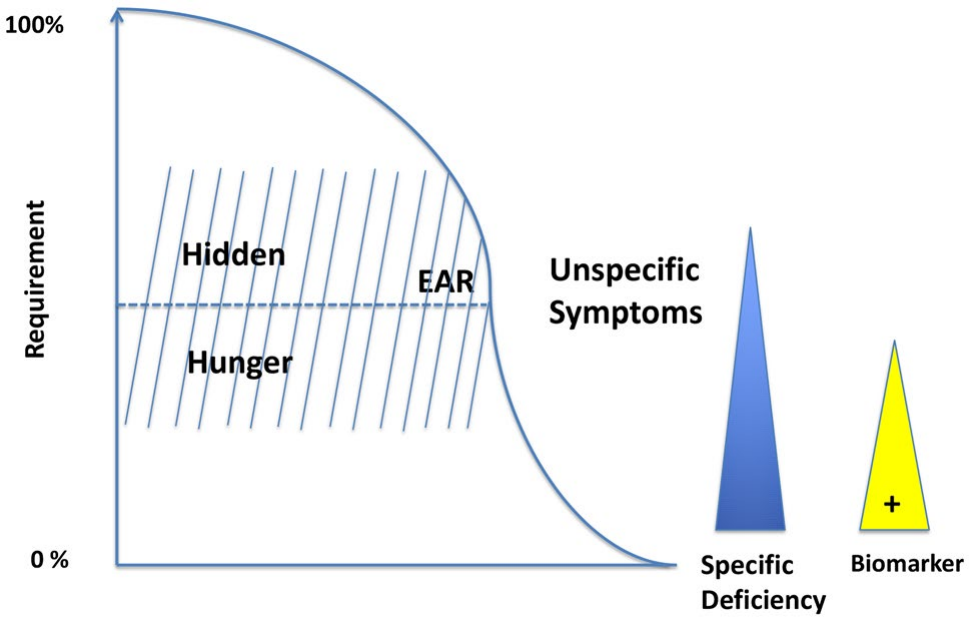
Deficiency as an end stage disease and hidden hunger. The range around the EAR is the amount of micronutrients that is adequate or inadequate depending on age and individual needs. In this range, the hidden hunger can be defined

Hidden hunger describes a condition in which there are neither clear clinical symptoms nor significant changes in blood levels of individual micronutrients. A clinically visible deficiency, such as scurvy or rickets, will only become apparent when lack of one or more micronutrients has been present for sufficient time. However, these diseases are end-stage and, during the progression, a number of changes (growth, immune system, metabolism) can occur due to an insufficient supply, which do not give a clear indication of the respective deficiency. Figure 1 shows the correlation between hidden hunger and supply. The EAR is the estimated average requirement and is obtained from large healthy populations to estimate the mean intake without signs of deficiency. Below the EAR value, the risk of deficiency increases, above it risk decreases. The same applies to the significance of the bioindicators (e.g., plasma levels), which are often only informative in the case of a pronounced deficiency. To define the recommendations, the twofold standard deviation of the mean value of this Gaussian distribution is added.

The most common deficiencies worldwide are in vitamins A, D, folic acid, iron, zinc and iodine [24]. A recently published study examining the micronutrient supply of healthy German children between 10 and 36 months shows that the supply of a number of micronutrients (vitamin D, iron, zinc, iodine) is in part significantly below the recommendations for this age group [79]. A study in 5 European countries (Germany, Belgium, Italy, Poland and Spain) concluded that the supply of calcium, iron, iodine, zinc, folic acid, vitamin D and B12 to children up to the age of 8 is suboptimal or inadequate [191]. These results confirm a European study that also describes an inadequate supply of vitamin D, folic acid and iodine in adolescents between 10 and 18 years of age [54]. For children in Germany in the age group 6–10 years, there is also a high prevalence of inadequate supply of folic acid, vitamin D, iron, zinc and iodine [90]. Although in these studies, household income was not considered, it can be assumed that the deficits are even more pronounced in families living in poverty. In its report, the WBAE^2^ [182] pointed out that data on the nutrition of poor families in Germany are virtually non-existent. These data are urgently needed, as it is the only way to make targeted interventions. It is well established that food has an inverse relationship between energy and nutrient density, and that energy-dense but nutrient-poor foods are comparatively inexpensive meaning that a more nutritionally favorable food choice is usually associated with higher costs per kilocalorie (kcal).

### Deficiencies in individual micronutrients can impair a child's physical development

Physical development is manifested by delayed length growth (short height for age), a condition known as stunting [104]. Stunting is a phenotype of malnutrition and is common worldwide, affecting up to 50% of children in different low-income countries. A study of children from poor households in the state of Brandenburg [15] concluded that children from low-income families were significantly shorter than children from families with higher socio-economic status. Children from households with two children were 0.5 cm shorter; children from households with four or more children were 1.8 cm shorter than single children. Children from households where the mother attended school for less than 10 years were 0.8–0.9 cm shorter than children from families with higher income [15]. "For example, in this study we found that children's height is very sensitive to the number of siblings. A 6-year-old child in Brandenburg who starts school is on average 1.8 cm shorter if he or she has three or more siblings. If the parents are unemployed, the disadvantage is even greater."

Crucially, growth retardation often cannot catch up after the age of five. As a result, the children are permanently physically limited, which, as large studies have shown, also has an effect on their possibilities of later pursuing a profession in which physical resilience is important [104].

The poor supply of essential micronutrients in the so-called ‘1000-day window’ (conception to the end of the second year of life) is considered to be the cause of stunting [134]. Studies in low- and middle-income countries have shown that a major cause is the preferential consumption of starchy foods because they are inexpensive and create satiety [60]. However, this is also the case in poor families in high-income countries. Especially at the end of the month, low-priced foods with low nutritional quality (few micronutrients), such as rice, pasta, potatoes, and few vegetables and fruits, are purchased. Foods with a favorable price/quantity ratio such as inexpensive and usually very fatty sausages or high-fat potato products are consumed in far greater quantities than fruit and vegetables. Here, the question of a healthy diet hardly arises, people buy what is inexpensive and what satiates them, as observed in case studies [103].

### Deficiencies in individual micronutrients can impair a child's cognitive development

**Fig. 2:**
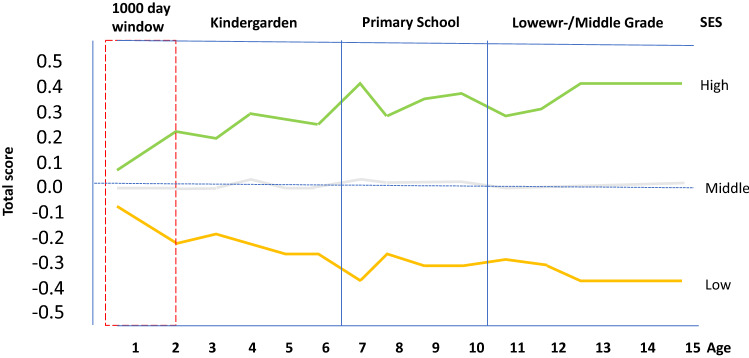
Achievement differences (composite index) in low-, middle- and high-SES families (modified from [153])

Using the German school system as an example, Skopek and Giampiero [153] investigated how large the known gaps in the cognitive development of children from low-socio-economic status (SES) households are in comparison to families with higher SES. They investigated how extensive these performance differences are before school entry and whether, as they expected, a convergence of the performance differences between the two groups can be observed again through schooling. The result surprised the authors: SES differences in cognitive development can be detected as early as 7 months after birth and persist throughout the entire school years. It is striking that the strongest difference in cognitive development becomes visible toward the second year of life and not, as the author expected, after the children have started school. However, this is precisely the 1000-day window during which cognitive development is particularly strongly influenced by nutrition [23]. Malnutrition and stress within the first years of life could explain the persistent development of cognitive differences despite the developmental opportunities at school (see Fig. 2).

In the above-mentioned study [153] with 57 children from Germany (German National Educational Panel Study), the strongest differences in performance were found in the first years of life (kindergarten), results that other investigators also confirm. Differences in language and reading test scores were most pronounced among children from different SES at kindergarten entry. While performance differences in the Skopek and Giampiero study in various cognitive indicators changed over time, the performance differences in reading and use of language remained even after enrollment and over the time of the entire schooling. The basis could therefore be a developmental disorder in the brain, which is triggered by both stress and malnutrition within the 1000-day window, but also in the following years. Differences in language development and in the use of words between children from families with low SES and those with high SES have been known for a long time [181]. For example, analyzing a (small) sample of infants in the United States, found significant SES differences in vocabulary and language processing when the children were eighteen months old; by twenty-four months of age, these differences had grown to a magnitude equivalent to 6 months of divergence in development [63]. Language processing is closely linked to the development of the hippocampus in the first years of life.

A large number of studies have now been able to prove that the growth of the brain can be disturbed in children who live in families with low socio-economic status [88] with malnutrition and stress having a negative impact on brain development during pregnancy and early childhood [107]. Children from low SES families are more likely to have structural brain development disorders than children from higher income families [23, 87, 158]. SES affects gray matter development (volume indicator) in different areas, especially in the hippocampus [77, 82, 122]. Children's early experience of parents who are stressed by their precarious situation (resources are insufficient to provide for the family) and pass this stress on to the children has an influence on cognitive development [122]. The ratio of income to basic needs rather than the educational level of the parents shows a positive association with hippocampal volume [42, 77].

The reduced hippocampus volume in childhood seems to be irreversible in adulthood and thus represents a permanent disadvantage [30, 156]. This may explain the permanent difference in performance observed by Skopek and Passaretti or also permanent poverty with all the associated negative influences on cognitive development.

If one looks at the developmental disorders or illnesses of children in Brandenburg, which were diagnosed in the context of school enrollment examinations, in relation to the income of the parents, a striking correlation emerges, especially in the area of cognitive developmental disorders. While 18.2% of children from families with a low social status are affected by speech and language disorders, this only applies to 4.3% of children from families with a high social status. Impairments in mental development were observed in 13.2% of children with low social status, but only in 0.9% of children with high social status [58].

### What can be done?

For a long time, the federal government has rejected every initiative, such as a request by the National Ethics Council or those of various political parties, to increase the funds for nutrition for households in receipt of unemployment benefits. On the one hand, the argument is used that this is a matter for the different states, which is quite true, or it is pointed out that there will be no change in the allowance as long as there are no corresponding measures for nutrition education at the same time. Yet there are many examples of how the problem can be successfully tackled, especially in times of pandemic but also beyond. There are enough examples in various countries that show that a moderate increase in funds leads to more food security and, above all, has a positive effect on the health and mental development of children in poverty [151]. We can learn a lot from the U.S. approach when it comes to migrants, who make up a large proportion of children in food poverty.

The impact of early intervention can also be seen in children's success at school (ages 2–5). Increasing parental income ($1,700 USD/year) shows a significant improvement in children's school performance compared to families without an income increase [81, 117].

Low food security is defined by the U.S. Department for Agriculture (USDA) when "households have reduced the quality, variety, and preference of their diets, but the quantity of food intake and normal dietary patterns have not changed significantly" [174]. It is precisely these changes that promote the development of obesity and as a consequence of involuntary preference of high energy and poor quality food. An increase of one USD/day per person in the food allowance has already had a significant effect on food choice and quality, leading to improved food security [8]. The funds were invested in more dairy products, fruit, vegetables and fish and less fast food.

The following demands are made, based on the available scientific work on the COVID-19 pandemic and the risk of food insecurity, especially among children [131]:

- Maintaining school meals for all children, but especially those from poor households.
- Detecting early-food insecurity and providing rapid intervention measures
- Targeting and rapidly providing assistance to households in poverty with children or pregnant women.

In contrast to other countries, Germany seems to lack not only the political will but also the sensitivity to perceive the problem and to eliminate the causes. To reiterate “what is not allowed to be, cannot be”, i.e., the problem seems that policy makers close their eyes and get on with it. The children are left with their health and a perspective for a better, i.e., healthier and productive, life on uncertain terrain.

## An overview of adolescent health and nutrition among low- and middle-income countries

### Context and epidemiology

There are approximately 1.8 billion young people of ages 10–24 years in the world representing almost 18% of the world’s total population [166]. About 90% of these youth are from low- and middle-income countries (LMICs) (Table 1) [135, 162]. The pre-adolescent and adolescent periods are a phase of rapid growth. These young people are going through a transitional period from childhood to adulthood with unique physical, emotional, and nutritional characteristics and requirements. For example, the expenditure of the total energy to support the growth during the pubertal period is more than the infantile period (3% vs 4%, respectively). Similarly, the need for the macro- and micronutrients, such as calcium, phosphorus, and iron are significantly different than both childhood and adulthood [119]. Although childhood nutritional status impacts the onset of puberty, adiposity, and linear growth, nutrition during the adolescence period impacts body composition, immune system development, and neurodevelopment [124].

Additionally, the adolescence period may also provide a short window of opportunity for catch up growth and correction of nutritional deficiencies rooted in infancy and childhood. An analysis of longitudinal growth data from the Health Orient Research in Transitioning Collaboration (Brazil, Guatemala, India, Philippines, and South Africa) and the Gambia showed that significant catch up height occurs in two periods, first between 24 months of age and mid-childhood and again between mid-childhood and adulthood [136].

**Fig. 3:**
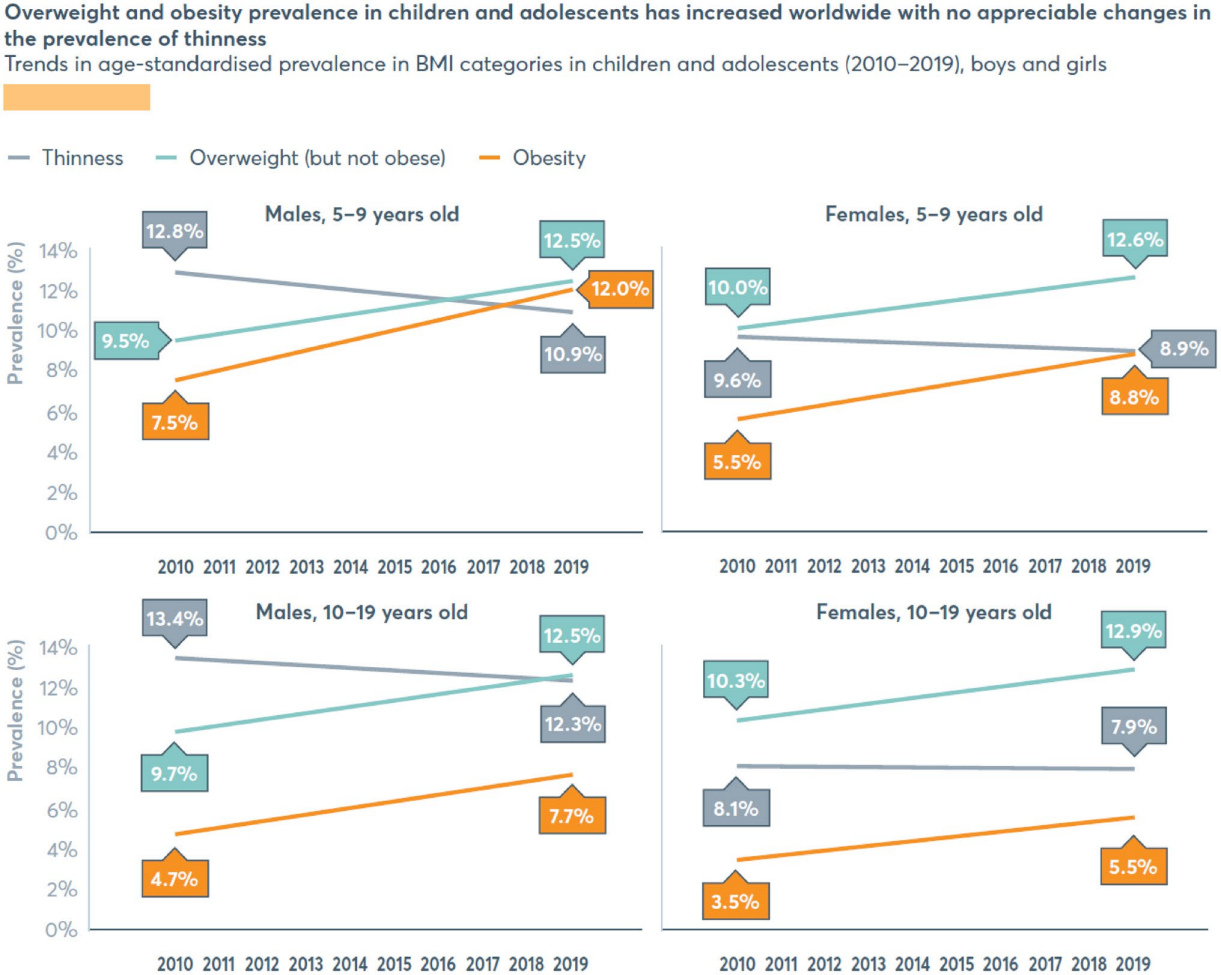
Trends in age-standardized prevalence in BMI categories in children and adolescents from 2010 to 2019 in boys and girls [70]

Despite the evidence of unique features and requirements of the adolescence period, this population is continually neglected. There are significant disparities in adolescent health between developed and developing countries. Due to continued focus on infant and child nutrition, their health and nutrition status has improved significantly in this global population in the last 50 years. Unfortunately, the same importance and focus have not been given to adolescent health and nutrition. The prevalence of moderate to severe underweight (< − 2 standard deviation BMI) is highest in South Asia where one in 5 girls aged 5–19 years are underweight [36]. Stunting, measured by height for age below 2 SD based on WHO/CDC reference values [183] is reflective of poor nutrition and adverse environmental stress. Pre-pregnancy stunting is considered a risk factor for small for gestational age babies and preterm birth; however, there are limited data available on the prevalence and outcomes of stunting in the adolescent population [95]. On the other hand, overweight and obesity are emerging health problems related to poor nutrition among adolescents in LMICs. The prevalence of obesity has increased from < 1% in 1975 to 5.6% in females and 7.8% in males aged 5–19 years in 2016, leading to a higher risk of non-communicable diseases, such as diabetes and cardiovascular diseases in adulthood (Fig. 3) [70, 120].

Micronutrient deficiencies add to the burden of inadequate nutrition and poor health of young people. For example, in adolescent girls, the requirement for iron intake increases to account for menstruation. Iron deficiency and resulting anemia account for more than 2500 disability-adjusted life years (DALYs) per 100,000 adolescents [5]. The prevalence of anemia in 15–19 years old girls in many LMICs exceeds the threshold of 40% at which it is considered a severe national public health problem [97]. In addition to anemia, iron deficiency can lead to weakened immunity, decreased cognitive function, and suboptimal growth in adolescents [100]. Iodine deficiency is present in 3.4% of young adolescent females of 10–15 years old and 4.6% of 15–19 years old females in the LMICs [36]. Limited data are available on the deficiencies of vitamins B, C, D, Zinc, and Selenium. Vitamin A deficiency is also prevalent in adolescent females, but the clinical significance of this deficiency is understudied [36]. Due to increased requirements and dietary variation, adolescents are also at high risk for calcium deficiency and related bone diseases [148].

### Adolescent pregnancies

Adolescent pregnancies further complicate the already comprised nutritional status of young girls and can lead to adverse outcomes for newborns. Approximately 21 million girls aged 15–19 years become pregnant and 12 million of them give birth every year [185]. Young age marriages and the social pressure to bear children soon after marriage are major contributors to adolescent pregnancies. The mean age of marriage in South Asia remains less than 20 years with one in 5 girls married before 15 years of age (Tables 2, 3) [164, 168]. There are wide variations of adolescent fertility rates within regions but overall, it is reported to be approximately 33 (number of births per 1000 girls ages 15–19) in South-East Asia, with Bangladesh having the highest adolescent fertility rate of 83 [185].

**Table 2:** Demographic profile of young people in South Asian Region

Country	Young people ages 15–24 (mid-2017)		Young people ages 15–24 (mid 2050)		Adolescent fertility rate ages 15–19
	Million	% of total population	Million	% of total population	
World	1189	15.9	1355	13.8	50
South Asia					
Afghanistan	7.6	21.4	12.1	17.6	78
Bangladesh	31.6	19.2	25.4	12.6	83
Bhutan	0.2	25	0.1	10	23
India	246.9	18.3	229.4	13.7	25
Nepal	6.2	21.1	4.4	13.2	71
Pakistan	38.4	19.3	49.2	15.8	44
Sri Lanka	3.2	15.0	2.5	11.4	15

The world youth 2017 Data Sheet [168]

**Table 3:** Mean age at marriage and percentage of ever married among females aged 15–19 and 20–24 [168]

Country	Mean age at marriage		Percentage ever married among women aged	
	Men	Women	15–19 age group^a^	20–24 age group^a^
Afghanistan	24.7	21.3	17.2	67.6
Bangladesh	25.5	18.8	45.2	84.6
India	26.0	21.3	15.4	66.8
Nepal	23.7	20.1	27.5	75.8
Pakistan	26.9	22.7	14.4	50.8

^a^A mean value was reported where two or more values were reported from different data collection sources

The increased requirement of protein, energy, and micronutrients during adolescence pregnancy coupled with increased needs for fetal development leads to a competitive environment with resulting suboptimal growth for adolescent mothers. A significant difference was noted between the height and body composition of adolescent mothers when compared to non-pregnant girls of the same age in rural Bangladesh [139]. Similarly, a study done in Brazil showed a significant difference in the height of pregnant adolescents relative to their counterparts [68]. Early pregnancy also places the mother at high risk of morbidity with eclampsia, systemic infections, and puerperal (postpartum) endometritis [185]. Young mothers are more likely to drop out of school and experience domestic violence within a marriage or partnership. More research is needed to explore the long-term health effects of early pregnancies in adolescent mothers.

Unfortunately, adolescent pregnancy’s adverse effects are not limited to adolescent mothers only. Adolescent pregnancies can lead to preterm births, obstetric complications, and a higher risk of neonatal and infant mortality. Maternal stunting can lead to cephalo-pelvic disproportion with high risk of intrapartum complications and obstructed labor. A recent study of a birth cohort from 5 LMICs (Brazil, Guatemala, India, Philippines, and South Africa) showed an increased risk of low-birthweight, preterm labor, low adult height, and higher adult glucose concentration in offspring of young mothers [59]. These poor pregnancy outcomes lead to a vicious cycle of poor nutrition and health status in future generations.

### Social determinants of adolescent health and nutrition

Adolescents' health and nutrition are deeply affected by individual, political, economic, and social factors. A comprehensive approach that includes the entire life course and social factors is of the utmost importance to better understand the deep-rooted problems and find the solution to improve health outcomes for this population. Many communities have cultural and gender norms that impact nutrition intake, physical activity, energy expenditure, and education, especially in female adolescents. Gender norms also affect early marriages and childbearing decisions. Economic factors in the developing countries affect the access to nutritious food and limit the buying capacity for low-income communities. Public health policies or lack thereof are important contributors to this global problem. Food choices, peer pressure, social media, and health literacy also impact the nutritional status of adolescents [121].

### Potential nutrition interventions

Only limited data are available to analyze the impact of nutritional and micronutrient supplementation in the setting of poor nutrition and micronutrient deficiencies. Iron supplementation has shown promising results in increasing the mean hemoglobin concentration among adolescent girls. A recent systematic review suggested that multiple micronutrients, zinc, iron, and iron-folic acid supplementation could significantly improve the serum hemoglobin concentration in adolescent female with pre-existing low hemoglobin [101]. The results from the calcium supplementation have shown mixed results. In a study from the Gambia, increased bone mineral content was noted after calcium supplementation (1000 mg/day), but no effect was noted on the linear growth. There is limited evidence on the benefits of protein and energy supplementation [52]. Zinc supplementation in pregnant adolescents improved low birth weight and preterm labor [101]. Studies are needed to evaluate the efficacy and long-term effect of multiple micronutrient supplementation in the preconception period for adolescents. There is an ongoing prospective, population-based, cluster-randomized trial of multiple micronutrient supplementation in the preconception period in young Pakistani girls aged between 16 and 24 years. The study will also evaluate the effect of culturally appropriate, bi-monthly educational sessions with focus on life building skills such as personal and menstrual hygiene, education about delaying early marriage and importance of good nutrition for health in those young girls [17].

### Recent challenges and way forward

COVID-19 pandemic has had devastating effects on the global economy and public health. It had an alarmingly high impact on maternal, child, and adolescent health and education in LMICs. About 12 million children are at risk of dropping out of school in South and West Asia and 1.2 million girls may not return to schools in East Asia [165]. Similarly, 250 million children are impacted by school closures due to the pandemic in sub-Saharan Africa [163]. It has not only impacted the education sector, but access to food, healthcare and contraceptive services have been severely compromised in the LMICs. In rebuilding health and education systems, due attention must be given to school-aged children and adolescents and their needs, especially those in marginalized populations.

In conclusion, greater focus and accountability are needed to provide the support during the adolescence periods, especially for females. A comprehensive approach including education, policymaking, addressing social determinants, access to reproductive services, and financial support can produce long lasting effects and can help to break the intergenerational effect of poor nutrition and health in future generations.

## The use of dietary supplements during pregnancy

Adequate micronutrient intakes are universally recognized as essential periconceptually and throughout gestation to ensure optimal maternal and child health [14, 25, 39, 45, 67, 137]. Indeed, micronutrient requirements are higher during pregnancy for almost all vitamins and minerals as outlined by the Dietary Reference Intakes [85]. If nutrient needs are not met during pregnancy, an increased maternal risk for transient disease risk exists during pregnancy, and risk for adverse birth outcomes for the offspring (e.g., low birth weight, preterm birth, congenital anomalies), as well as delays in developmental milestones, including delays in neurocognitive development.

To prevent dietary inadequacy in the U.S., most pregnant women (> 75%) use prenatal dietary supplements (DS) [13, 28, 89]. Data from the National Health and Nutrition Examination Survey (NHANES) indicate that the prevalence of a dietary supplement use during pregnancy is related to maternal age, trimester, income, and race and ethnicity, as well as having health insurance [28]. Older (35–44 years) pregnant women report a higher prevalence of use (88%) than younger (20–34 years) women (75%). Prevalence of use increases linearly to family income, for both any DS use and for prenatal DS products, and non-Hispanic white women have a higher prevalence than other race ethnic groups (9). The majority of products used are reported to be recommended by a healthcare provider, and contain micronutrients and are classified as multi-vitamin-minerals (i.e., ≥ 3 vitamins and at least 1 mineral; MVM). However, there is variation in the number and amounts of nutrients in these MVM products and prenatal supplements can also differ in composition based on if they are sold over the counter or by prescription [145]. In general, the DS products used by pregnant women exceed the Recommended Dietary Allowances for most vitamins, iron, and zinc; however, are lower than the RDA for choline, calcium, iodine, magnesium, phosphorus, and selenium [89]. While diet quality (from foods) during pregnancy, as assessed by the Healthy Eating Index (HEI; range of scores from 0 to 100), is higher during pregnancy (HEI = 63) than that of similarly aged non-pregnant women (HEI = 54) [53], most women fail to meet dietary recommendations for key nutrients, unless a DS is used.

Total usual nutrient intakes (i.e., estimates combining foods, beverages, and dietary supplements) of pregnant women from the NHANES [13] and National Environmental Influences On Child Health Outcomes (ECHO) Consortium [146] studies both suggest a very low risk of dietary inadequacy for thiamin, riboflavin, niacin, vitamin B12, and phosphorus. Given that the majority of pregnant women use nutrient-containing dietary supplements and the amounts contributed by the products alone, the risk of dietary inadequacy is lower for many nutrients based on total intakes (from both food and dietary supplements) when compared with intakes from food alone for most micronutrients. For example, with the use of DS, many women were at risk for low intakes of magnesium, iron, zinc, potassium, calcium, choline, magnesium, phosphorus, and selenium and vitamins A, B6, B9, C, D, E, and K. Both NHANES and ECHO data confirm that most pregnant women consume too much sodium from the diet alone, and only negligible amounts of sodium are in DS products. Very few pregnant women have intakes above the tolerable upper intake level (UL) from food and beverages alone; however DS use increases the proportion of women with intakes above the UL (i.e., potentially at risk of adverse effects due to excessive intakes) of some nutrients, especially iron and folic acid. While, DS use attenuates the risk of inadequacy for some (though not all) micronutrients, their use places many women at risk of excessive intake for iron and folic acid [13, 146].

A few other studies using national data focused only on supplements containing folic acid, iron, or iodine during pregnancy and before pregnancy [28, 40, 65, 76]. For example, pregnant women who use supplements containing these nutrients, the average amounts exceed or approach the UL, just from the DS alone: folic acid at 817 mcg (UL = 1000 µg) and iron at 48 mg (UL = 45 mg) per day. These data are suggestive that the amounts of these nutrients may be too high in some prenatal products. Canadian guidelines have suggested that 400 µg of folic acid supplementation is adequate for most non-high risk pregnancies (16), but the products used in the U.S. continue to be well above this recommendation [145]. Serum and red blood cell concentrations of folate are higher among those using in iron DS, but folate biomarkers are still lowest in the first trimester of pregnancy [28] and this is the critical time period for prevention of neural tube defects [115, 171].

Biomarker data also suggest that many reproductive aged and pregnant women have a risk of iron and iodine inadequacy, both of which have the potential to cause irreversible neurocognitive defects and behavioral changes in the child. Iron deficiency and anemia are more common during pregnancy when women do not use DS [40]. The American College of Obstetricians and Gynecologists recommends the use of iron DS during pregnancy to reduce the risk of iron deficiency anemia [2]. Similarly, the American Thyroid Association recommends that all women who are pregnant use a dietary supplement with a minimum of 150 µg of iodine a day [6, 138]. While most prenatal supplements contain the recommended amount of iodine [65, 145], their use is low among pregnant women (20.4% consuming 116 µg/day from supplements on average) [65, 76, 132]. Urinary iodine concentrations suggest that many reproductive aged and pregnant women have risk iodine inadequacy [65, 76]. A primary limitation of the databases used to assess intakes in NHANES and ECHO is a lack of data on iodine values in foods and beverages, given that iodine is critical for fetal brain development [84].

### Summary

Pregnancy is a critical window of opportunity where optimal nutrition is critical for health. Disparities in risks of inadequate or excessive intake from large, national data sources suggest a need for strategies to improve the micronutrient intakes during pregnancy, and dietary supplements have the potential to help women meet needs, but a tailored approach to reformulate products consumed during this life stage remains.

## Midlife tune up for women: a critical point for risk screening and lifestyle modification to achieve healthy longevity

Females’ life-stages correlate tightly with the rise and the decline of estrogen. The surge of estrogen at puberty prepares females for childbearing [18] and at the same time it has been shown to also protect against cardiovascular diseases. At around 50 years of age, an acute reduction of estrogen production (menopause) takes place [98], precipitating menopausal symptoms, such as hot flashes, mood changes, slowed metabolism and a number of undesirable health sequelae which could require medical attention. Menopause signifies a critical intervention point for risk screening and individual lifestyle modification for managing these issues and ensuring healthy longevity.

### Multiple indicators of health deteriorate sharply at menopause (see Fig. 4)

**Fig. 4:**
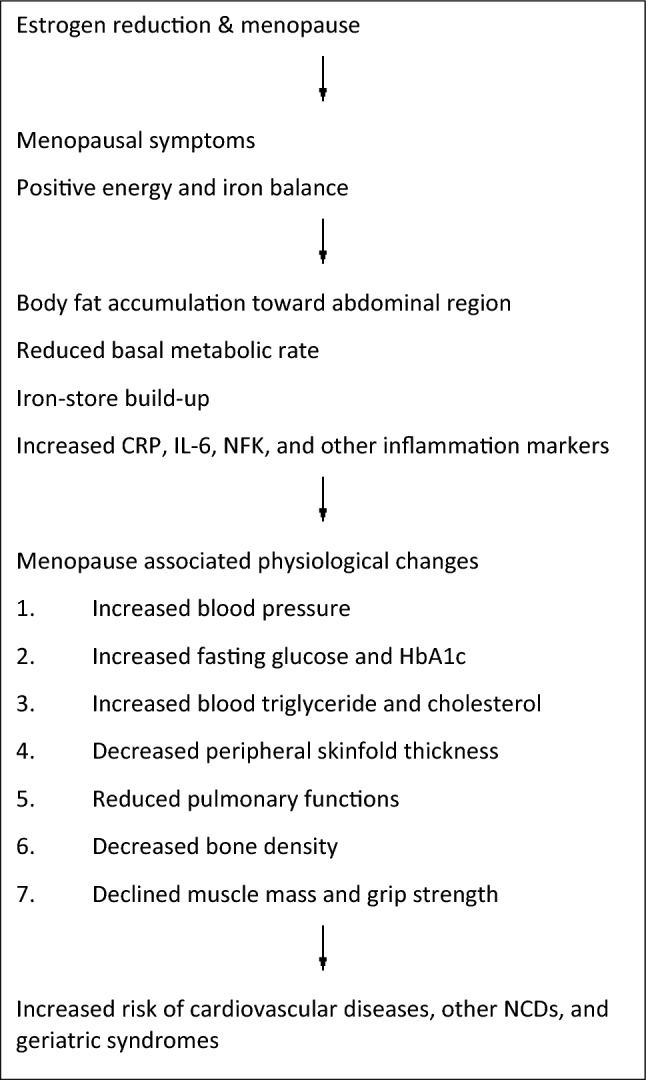
Menopause-associated physiological changes

Estrogen has been recognized as an agent which protects women against the risk of many non-communicable diseases (NCDs) [86]. Descriptive epidemiological studies have shown very clear phenomenon of lower levels of cardiometabolic risk factors before menopause compared to men and a sharp rise afterward [99]. Sometimes we even observe a cross over in these risk factor profiles between genders. These cardiometabolic risk factors include abdominal fat accumulation [34, 160] and elevation of blood pressure, fasting blood glucose, triglyceride, cholesterol, and uric acid [34, 99]. In line with the decrease in estrogen, research has observed systematic body composition changes which affect functional performances, including muscle [111] and bone loss [93].

After menopause, body fat not only accumulates due to a positive energy balance, but it is also surprisingly distributed primarily in the abdominal regions [160]. Compared to subcutaneous fat, abdominal fat releases more inflammatory cytokines such as C-reactive protein (CRP), interkeuken-6 (IL-6), and tumor necrosis factor α (TNFα) [127]. The decrease in estrogen also compromises the anti-inflammatory effect on IL-6, Cox-2, etc. [127]. Another highly associated phenomenon concerns iron storage which is very low in pre-menopausal women due to the regular blood loss from menstruation [126]. Iron is a well-known pro-oxidant [66], and low levels of iron in the body minimizes oxidative stress and thus may lower the risk or retard inflammatory processes. Once blood loss stops at menopause, body iron stores in women will quickly build up [126]. All of the afore-mentioned would situate the body in increased oxidative and inflammatory states, which may induce or exacerbate menopausal symptoms and hasten multiple aging.

### “Lifestyle medicine” provides a holistic way to tune up post-menopausal physiology

When a woman loses her estrogen protection at menopause, her usual pre-menopausal lifestyle may no longer be able to maintain her healthy body and satisfactory functional performance. If there are no changes made at this point on, her physiology will rapidly roll downhill [34, 93, 99, 111, 160], i.e., increased risks of metabolic syndromes; weakened muscle, lung function, and bone. And in the long run it may lead to cardiovascular and other non-communicable diseases and earlier onset of geriatric syndromes. Since all organs and systems are exposed to this post-menopausal inflammatory state, system or organ aberration may take place one by one and current poly-prescription medicine may be the sole antidote. To curb the development of such cascading processes, the better approach would be to practice “lifestyle medicine”. That is to provide to the body with sufficient amounts of natural diet-related antioxidants and anti-inflammatories along with all of the needed nutrients including minerals, vitamin D, and protein particularly for bone and muscle health.

### Randomized controlled trials demonstrate the efficacy of lifestyle and dietary approaches on total wellbeing

In recent decades, several landmark studies showed the efficacy and potency of a healthy lifestyle approach on controlling or reversing development of cardio-metabolic diseases. The Diabetes Prevention Program [94] found that a minimum of 7% weight reduction and maintenance combined with a minimum of 150-min brisk walking or equivalents per week was able to reduce diabetes incidence by 58% for high risk individuals. Its effect outweighed the pharmaceutical (metformin) group. It may be even more beneficial to provide higher than basal levels of relevant dietary nutrients to the susceptible. The Dietary Approaches to Stop Hypertension (DASH) trial [144], a 3-month dietary provision combining multiple beneficial dietary factors (more vegetable, fruit, whole grains, nuts and seeds, low-fat dairies, plant protein foods and n-3 fatty acid rich fish; and less red meat and sweets) exhibited a significant blood pressure lowering effect which was equivalent to that of one antihypertensive medicine for hypertensive patients. Furthermore, the health effect of the Mediterranean diet has been trialed recently in a large-scale study (PREDIMED trial) [64] and showed protection on cardiovascular events, depression, and cognitive decline. Prospective studies investigating dietary patterns with either DASH or Mediterranean diets also showed long-term effects [33] on maintaining weight loss, preventing kidney stone formation, and lowering the risk of some cancers, diabetes, heart failure, and delaying chronic kidney disease and Parkinson progression. Data derived from a healthy Taiwanese Eating Approach (TEA diet) [37] featured a higher frequency of consuming tea, fish, vegetables, and fruit; and less red or processed meat, preserved vegetables, and sweets is associated with a lower risk of all-cause and cardiovascular disease mortality.

### Healthy diet ensures lifelong optimal health

The studies described above as well as others show that protective dietary patterns for various NCDs share similar features. All of them mention healthy dietary patterns providing sufficient essential nutrients and large quantities of anti-inflammatory substances, fiber, plant protein, and cardiovascular protective electrolytes (potassium, magnesium and calcium) along with a fatty acid composition rich in unsaturated, and low in saturated. The same diets also fulfill dietary requirements for preventing osteoporosis (such as sufficient levels of calcium, vitamin D and isoflavones) [31] and sarcopenia (such as protein) [16, 149]. It appears that these plant-based dietary patterns provide multiple beneficial components which are essential for optimal health and preventing various diseases occurring at different life stages.

**Fig. 5:**
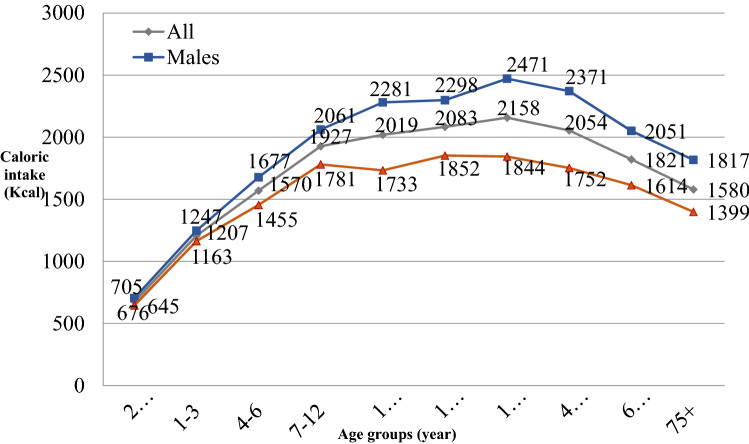
Sex and age-specific level of average daily caloric intakes, Nutrition and Health Survey in Taiwan [189] (unpublished data from the author)

Survey data shows that very older age is associated with less caloric intakes (see Fig. 5) while the percentage of abdominal fat situates at the highest point in the whole lifespan (5). Inadequacy (low levels) of multiple nutrients has been documented for geriatric outpatients [190]. The dilemma is that older individuals have the inability to maintain usual levels of physical activity which lowers energy expenditure and compromises appetite and the opportunity to use and to build muscles. Lack of appetite prevents obtaining sufficient nutrients and anti-inflammatory substances from foods, creating a vicious cycle.

### Midlife is a critical time point for women to tune up

Women at midlife experience discomfort associated with menopausal symptoms and risk elevation of multiple NCDs. It is a crucial time point to re-examine whether a woman’s lifestyle meets the new physiological needs and for her to make adaptive changes to reach a new balance required for ensuring a healthy aging process. A tailored healthy dietary plan can be made according to one’s risk factor profile. To live an active life, consuming sufficient amount of nutrient dense foods is pivotal for healthy aging, starting at or before menopause.

## Nutrition and health disparities among low-income women in a high-income country, the solutions offered through federal programs and the gaps that remain: the U.S. as a context

High-income countries throughout the world experience high chronic disease burdens that are greater among the low-income groups within those countries. To support food access and close the health gaps among low- and high-income groups, many countries offer public health sustenance programs. Women are especially at risk as they often earn less compared to men and more often head single-parent households with children [29]. The U.S. provides an ideal context to examine nutrition and health disparities, and federal programs directed to ameliorate food security (the access to enough nutritionally adequate and safe food for an active healthy life) [55] poor dietary intake and quality, and chronic disease among low-income women.

Heart disease, cancer, diabetes, and obesity are among the top causes for death in the U.S. [4, 7]. Changing the behaviors of daily living, specifically poor dietary habits, a leading risk factor, may prevent many of these premature non-communicable disease deaths [178]. Advice describing what to eat and drink to promote health and meet nutrient needs, is provided through the Dietary Guidelines for Americans (DGA) and used to direct interventions [173]. This national nutrition policy translates current scientific evidence into dietary recommendations that address population nutrient concerns (i.e., underconsumption of calcium, potassium, vitamin D, and fiber and overconsumption of sodium, saturated fat and added sugars) and corresponds to U.S. food culture and preferences. However, the actual nutritional quality of dietary intake of U.S. adults compared with the DGA is also ‘poor’ despite a plentiful food supply as indicated by a mean score of 59 out of 100 on the Healthy Eating Index (HEI), translating to a F (failing grade) on a letter gradescale [173]. The HEI is a measure of dietary quality that can be applied to evaluate how well a group of foods aligns with key recommendations of the DGA.

Having a low-income presents an additional nutrition and health risk compared with the already poor dietary quality of all U.S. adults. When a family’s total income is less than the Federal Poverty Guideline for the family size and composition, the family is classified as “in poverty” [172]. A family of four was in poverty in 2022 when annual income was below $27,750 [62]. About 11.4% of people in 2020 were in poverty (only slightly higher than pre-pandemic 2019 estimates of 10.5%) despite a wealthy economy [152] and about 15.3% of U.S. households were classified as having incomes less than 1.3 of the income-to-poverty ratio [152], a level qualifying for federal food assistance and often termed “low-income”. Food insecurity, defined as the limited or uncertain availability of nutritionally adequate and safe foods or limited or uncertain ability to acquire acceptable food in socially acceptable ways, may be a more direct indicator of nutritional risk as it differentiates among those with low-incomes who still have sufficient access to food and those who do not [41, 55, 73]. Food insecurity impacted 10.5% of U.S. households in 2020 (unchanged from 10.5% pre-pandemic 2019 estimates) [41]. Rates of both low-income and food insecurity are higher for women compared with men and for younger compared with older adults [41]. Having less income for all expenses and less access to food because of resources may limit food choices to a smaller and more economical selection of foods along with other factors optimized in purchasing like, taste and culture preferences, preparation time and equipment, food storage options, and health of the food [133].

Dietary quality is lowest for those with incomes below 1.3 of the poverty income ratio and in food insecure compared with food secure households [80, 142]. Even among only low-income adult women eligible for food assistance, food insecurity was linked with lower dietary quality compared with food secure adult women (HEI of 41 vs. 46) [142]. Food insecure adults consumed less fruits, vegetables, and dairy compared with food secure adults [78, 188]. Similarly, those with the lowest compared with higher incomes had lower intakes of dietary components to increase like total vegetables, and dark green and orange vegetables and legumes, and whole grains and higher intake of dietary components to decrease like solid fats, alcoholic beverages and added sugars [80]. Nutrient disparities were also present among groups by food security and income status. Fewer women with incomes below 1.3 of the poverty income ratio met the Estimated Average Requirements for calcium, folate, magnesium, and vitamins A, C, D, and E compared with higher income women [12] and similarly, fewer food insecure women met the Estimated Average Requirement for magnesium and vitamins B6, C, and D, compared with food secure women [43] and also had lower intakes of calcium, zinc, and vitamins A and B6 [78]. Accordingly, several risk factors and chronic health outcomes are also more prevalent in low-income and food insecure groups including poor or fair health, physical function, glycemic control, greater hypertension, cancer, cardiovascular and heart disease, diabetes, and obesity [19, 56, 74, 105, 159, 177].

**Fig. 6:**
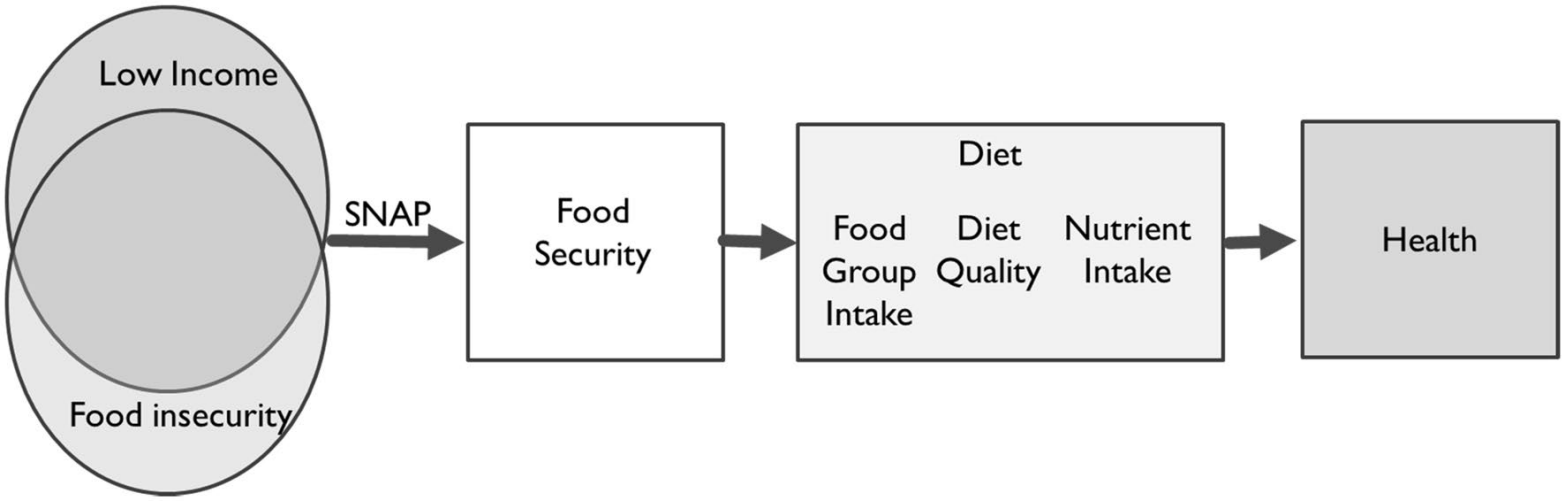
Hypothesized impacts of U.S. federal food assistance through the Supplemental Nutrition Assistance Program (SNAP) among low-income and food insecure groups on food security, diet and health

Federal food assistance includes several programs designed to intervene and improve food security and food access (see Fig. 6 above). The main U.S. federal food assistance program is the Supplemental Nutrition Assistance Program (SNAP) which provides benefits to purchase food for those at 1.3 of the poverty income ratio [176] and was used by about 11% of U.S. households in 2018 [106]. The average monthly benefit for a family of 4 is $638 in 2022 [175]. Evaluation of the effectiveness of the program presents many challenges to investigators such as the ethical application of experimental study designs, selection bias where those who are in the most difficult situations enroll most prevalently, sliding benefits, and miss-reporting [9]. However, SNAP has been evaluated in several studies and shown to improve food insecurity; participants are between 14.9 and 36.6% less likely to be food insecure than non-participants [48, 72, 75, 118, 123]. Furthermore, SNAP expansions occurred alongside prevalence estimates that flatlined from 2019 to 2020, when a spike in prevalence was expected due to the coronavirus pandemic [41]. Because of SNAP effectiveness to improve food insecurity, improvement in dietary intake, dietary quality and health outcomes may be possible as well (Fig. 6), yet these outcomes have been more challenging to determine [9, 20, 32]. A review, including research based on rigorous designs and national samples showed that SNAP participants had either similarly low or significantly lower dietary quality compared with non-participants [9]. Evaluation of health outcomes showed currently having poor health is linked with SNAP use but SNAP access may reduce future health risks [20]. SNAP is also linked to obesity in women but also to lower health care costs [179]. Nutrition education through SNAP-Education (SNAP-Ed), a non-entitlement program providing nutrition and budgeting education to households who qualify for SNAP, also improved household food security by 25% from baseline to 1-year follow-up in a randomized controlled trial of SNAP-Ed, but dietary quality and intake were not improved [57, 140, 141]. A hypothesis to explore in future studies is the potential dietary improvements of other family members, and especially children, due to SNAP and SNAP-Ed as adults in the household may reserve food considered more healthful for children in the household. The lack of evidence for diet quality and intake outcome improvements presents an opportunity for further evaluation through creative study designs and analysis to overcome the barriers in evaluation and measurement error of the long-term behaviors of dietary intake and their manifestations as health outcomes. Program expansion also has potential to magnify effects on outcomes and address the goal to eliminate U.S. food insecurity and the related dietary and health disparities.

## Recent developments in maternal, adolescent, and child micronutrient interventions in low- and middle-income countries

The effects of the COVID-19 pandemic are further dragging us off course as we try to reach global nutrition goals. An estimated 155 million additional people worldwide are pushed into extreme poverty due to the pandemic, and those who are obese or have other chronic and/or diet-related diseases are more vulnerable to COVID-19 infection, hospitalization and death [49]. Within 3 years, the pandemic will cause an additional 3.5 million stunted children (children who are too short for their age), 13.6 million wasted children (children who are too thin for their height), and add 283,000 deaths among children under 5 years of age, due primarily to malnutrition in low- and middle-income countries (LMIC) [125]. Climate shocks, conflict, and COVID-19 all increase the unavailability and cost of nutritious foods, which, combined with low incomes, increase the unaffordability of healthy diets. Today, three billion people around the world cannot afford a healthy diet, a phenomenon associated with higher levels of moderate and severe food insecurity and malnutrition [61].

Addressing the full life course provides a strong framework to focus attention to human nutrition’s challenges. For example, poor nutrition often starts in utero and extends, particularly for girls and women, well into adolescent and adult life and into the next cycle of pregnancy, ad infinitum [1]. Policymakers have recently placed a great deal of emphasis on the 'first 1000 days' as a window of opportunity to influence a child’s health outcome [44]. The first 1000 days correspond roughly to the time from conception to 2 years of age. Some emerging interventions, such as preventive small-quantity lipid-based nutrient supplements (SQ-LNS) for children aged 6–23 months, have shown positive effects on child growth and have recently been recognized with strong evidence to be an effective strategy to prevent malnutrition in low resource settings [50]. This is in addition to other well-known interventions, such as micronutrient powders (MNP), which have been scaled up since 2004, initially as part of the Tsunami recovery program, are still going strong today, reaching more than 16 million children through the United Nations Children's Fund (UNICEF) [167]. However, implementation issues remain to be addressed, namely access and use of MNP, harmonization of formulations, and questions about program performance in general [130, 143, 147].

There has been a great deal of emphasis on early childhood nutrition; however, adolescence (10–19 age group according to the WHO) is also an important stage of life that has lifelong and intergenerational implications. Although the world is home to 1.2 billion adolescents, of which 90% live in LMIC [128], this age group has been neglected in national and global plans and policies. The adolescent years also provide opportunities and risks, when consuming or missing healthy nutrition. During adolescence, the height velocity is second only to the first 2 years of life [129]. Changes in brain structure, function, and connectivity mark adolescence as a second period of opportunity to develop new relationships with adults and peers, and to discover one's identity. It is also a period of resilience that can ameliorate childhood setbacks and lay the foundations for a thriving future over the remaining life course [26]. Much remains to be done to put adolescent nutrition at the center of global plans and policies, and the recent Lancet series on adolescent nutrition represent a major step in the right direction by highlighting the effect of nutrition on adolescent growth and development, the role the food environment has on food choices, and which strategies and interventions might lead to healthy adolescent nutrition and growth [26].

In terms of maternal malnutrition, it remains a global challenge, especially in LMIC where underweight, micronutrient deficiencies, anemia and low birth weight (LBW) are a threat to the health and survival of women and children [180]. Among women of reproductive age, the prevalence of low body mass index has been reduced, but their stunting prevalence remains high [180]. Data on micronutrient deficiencies are limited, particularly for women, but research has shown improvements, specifically in vitamin A status [180]. The prevalence of anemia and zinc deficiency remain elevated and anemia has been defined as an intractable problem that requires innovative solutions [35]. Thirteen years after the first Lancet series on Maternal and Child Under-nutrition, the latest series published in 2021 reviewed the global agenda for tackling undernutrition. Evidence has increased for the effectiveness of antenatal multiple micronutrient supplementation (MMS) in reducing the risks of stillbirth, babies born small for gestational age (SGA), and LBW [27]. The 2016 WHO antenatal care guidelines which were released before the meta-analysis by Smith et al. [154], did not universally recommend MMS, stating that “there is some evidence of additional benefit … but there is also some evidence of risk” [157, 184]. However today, the scientific community has met all WHO’s concerns about the lack of evidence and potential risk of MMS. Indeed, compelling scientific evidence shows that taking MMS during pregnancy reduces the risk of maternal anemia and reduces the likelihood that a child will be born with LBW and SGA [27, 154, 157]. Anemic and underweight women benefit even more from MMS and have a reduced risk of infant mortality and preterm births than mothers taking only iron and folic acid (IFA) [154]. In 2021, MMS was included in the WHO Essential Medicines List to facilitate implementation research and programs in countries [114]. Implementation research around supply readiness and demand generation for MMS is currently ongoing in more than 25 countries across the globe.

Finally, supplementation is not the only solution to address hidden hunger (micronutrient deficiencies) and chronic malnutrition. Whole foods, such as eggs can play a critical role in the fight against malnutrition; one egg/day has been shown to reduce stunting in several contexts [10, 83].

Finally, improving the diets of the world’s poor is a complex and long-term undertaking requiring transformations of food systems. The ongoing COVID-19 disruptions and climate change will continue to exacerbate global malnutrition. In addition, raising incomes which can allow access to affordable, safe, desirable, and nutritious food are long-term goals and will not eradicate malnutrition today. Evidence-based large-scale programs (supplementation, fortification, biofortification) as well as social safety net programs, are necessary to improve affordability, nutritional status, and health outcomes, and to ensure that no one is left behind.

## Conclusion

When considering women’s ability to age healthfully, it must be more than just the narrow focus on reproduction and hormonal wellness. Physiological parameters associated with age-related decrements specific to women are part of the broader spectrum of opportunities and benefits to address gender-driven structural/functional distinctions and the role of nutrition.

There is a fundamental role for baseline recommendations for daily levels of vitamins and minerals, but also documented advantages ascribed to the intake of nutrients beyond simple adequacy, and attention to and education about should be part of the landscape to ensure consumption as part of a diet plentiful in these components. Diets need to be rich in proven constituents to achieve true ‘healthy ageing’ by supporting, for example, bone health, lactation, hormonal fluctuations and menopause [186].

Women, though with statistically significant longer lifespans, at least in the developing countries, must be recognized for their unique differential probability of some chronic diseases, such as their greater risk for bone and muscle loss. Nutrition focused on different cultural, socio-economic, and life-course communities, as well as applicable at all stages of growth and development must not be a one-size-fits-all approach. To be relevant to the individual, there needs to be a degree of ‘precision’ applied such that a generic singular nutritional template does not result in unwanted and unhealthy overages with potential concomitant health decrements often of more immediate concern than the under-nutrition scenario that prompted the original recommendation.

There must be access to nutritional foods, and the information to grasp and put into practice proven nutritional opportunities. Nutrition science is moving toward understanding optimal nutrition in the broader context of dietary patterns, and nutrient intake must consider the complexity of interactions involving not only adequate nutrition but also the interplay with social, behavioral, environmental, community dynamics, and many other lifestyle factors. *Optimal is greater than the sum of the merely sufficient*.

## Footnotes

1 Bundesministerium für Ernährung und Landwirtschaft (BMEL).

2 Wissenschaftlichen Beirats für Agrarpolitik, Ernährung und gesundheitlichen Verbraucherschutz (Scientific Advisory Board for Agricultural Policy, Nutrition and Consumer Health Protection).
